# Towards Improved Linkage of Disaster Risk Reduction and Climate Change Adaptation in Health: A Review

**DOI:** 10.3390/ijerph15040793

**Published:** 2018-04-18

**Authors:** Nicola Banwell, Shannon Rutherford, Brendan Mackey, Cordia Chu

**Affiliations:** 1 Centre for Environment and Population Health, School of Environment, Griffith University, Brisbane 4111, Australia; c.chu@griffith.edu.au; 2 Centre for Environment and Population Health, School of Medicine, Griffith University, Brisbane 4111, Australia; s.rutherford@griffith.edu.au; 3 Griffith Climate Change Response Program, Griffith University, Gold Coast City 4222, Australia; b.mackey@griffith.edu.au

**Keywords:** disaster, health, climate change, disaster risk reduction, climate change adaptation, integration, synergy, linking

## Abstract

Climate change and climate-sensitive disasters significantly impact health. Linking Disaster Risk Reduction (DRR) and Climate Change Adaptation (CCA) is essential for addressing these ever present, complex and increasing risks. Recent calls have been made to build these links in health. However, there is a need to clearly articulate why linking DRR and CCA is important in health. Furthermore, little is known about how DRR and CCA should be linked in health. By extensively examining relevant literature, this review presents the current state of knowledge of linking DRR and CCA in health. This includes the potential for maximising conceptual synergies such as building resilience, and reducing vulnerability and risk. Additionally, technical and operational synergies are identified to link DRR and CCA in health, including: policy, Early Warning Systems, vulnerability and risk assessment, health systems strengthening, infrastructure resilience, disaster preparedness and response, and health impact pathways. Public health actors have a central role in building these links due to their expertise, work functions, and experience in addressing complex health risks. The review concludes with recommendations for future research, including how to better link DRR and CCA in health; and the opportunities, challenges and enablers to build and sustain these links.

## 1. Introduction

Climate change and climate-sensitive disasters, such as those resulting from hydrological, meteorological, biological and climatological hazards have significant and increasing impacts on human health [1,2,3]. Climate change and climate-sensitive disasters impact health through common direct and indirect pathways potentially resulting in increased risk of death, disease and injury, as discussed in Banwell et al. [4] in this special issue. An increase in well-planned, effective and appropriate adaptation and risk reduction is necessary over the short-, medium- and long-terms to address climate change and climate-sensitive disaster risks [1,5,6,7]. Increasing health action in two broad management paradigms, Disaster Risk Reduction (DRR) and Climate Change Adaptation (CCA), under the umbrella of sustainable development, has a significant role to play in this process. This is reflected in key international DRR and CCA policies, and comes as a result of the ever-growing role of health in these contexts [6,8,9,10,11]. This comes partly as a result of the knowledge that health co-benefits of mitigation and adaptation provide a ‘health lens’ and create a relatable and meaningful frame of reference for engagement on climate change issues [8,9,12]. Additionally, the growing understanding that appropriate adaptation can greatly reduce the health burden resulting from climate change and disasters [13,14,15,16] has seen the recognition of the role of health in DRR and CCA increase. Here, health is used to broadly refer to the government and non-government actors on international, national, sub-national and local levels with a role in public and primary health care, as has been used in previous literature (e.g., [13,14,17,18,19]).

A large body of research has suggested that building links between DRR and CCA in the context of sustainable development is essential and important to ensure a robust approach to dealing with climate-sensitive disasters and their associated risks [2,8,20,21,22,23]. Linking DRR and CCA strategies makes operational sense as it will enable countries to enhance coherency and synergies between DRR and CCA approaches, as well as maximize efficiency in terms of the human, technical and financial resources used to reach common goals, thus reducing the use of resources in duplicated institutional structures and implementation [2,8,21,23,24,25,26,27]. Additionally, linking these two approaches makes technical sense, enabling the sharing of expertise, knowledge, lessons and tools between the two fields, thus increasing the efficiency and effectiveness of risk reduction and adaptation particularly through the consideration of long-term risk and adoption of long-term adaptation options [2,8,21,23,24,25,26]. For example, linking DRR with CCA and climate change science has been recognized as necessary for improving DRR strategies, understanding risk, dealing with uncertainties, addressing increasing vulnerability as a result of climate change, and reducing the potential for maladaptation [2,25,26,28,29,30,31,32,33,34,35,36,37].

An opportunity for improving the links between DRR and CCA in the context of sustainable development was identified with the overhaul of several key international policies in 2015 [24,38,39]. The Sendai Framework, Paris Agreement, 2030 Agenda for Sustainable Development and Sustainable Development Goals, and the New Urban Agenda were created in a time of increasing coherence between international policies [10,16,24,40,41]. They emphasize the need to work towards linking DRR and CCA in practice [10,16,24,40,41]. Since this time, there have been efforts through international platforms such as the Global Platform for Disaster Risk Reduction [42] and the United Nations Framework Convention on Climate Change (UNFCCC) technical expert meetings on adaptation [26], both in May 2017, to build an understanding about building these links in national policies and various sectors. However, health was not discussed, illustrating that there is limited recognition of the importance of building links in health.

Health is an important end point for both climate change and disasters, and the recognition of the role of health is growing in both DRR and CCA internationally [10,13,14,16,40,41,43,44]. Health has been highlighted as a cross-cutting issue in DRR, CCA and sustainable development [17]. There have been some appeals for greater links between DRR and CCA strategies in health, particularly since 2015 [17,18,40,45,46]. To support these requests there is a need to clearly articulate why linking DRR and CCA is important in health. Furthermore, there are significant knowledge gaps around how to develop these links. There is a need to draw this literature together and summarise what is currently known to strengthen the advocacy for building links in health and identify areas for future work. Therefore, this article reviews relevant literature to provide a synopsis of what is currently known about linking DRR and CCA in health. It aims to provide a strong and coherent rationale for building links in health, discuss what the current literature conveys about how these links can occur, as well as highlight current knowledge gaps. In doing so it intends to serve as the foundation for driving future work on how these links can be formed and advanced.

Here the term ‘linking’ is used to describe the bridges and connections between DRR and CCA in policy and implementation that have been commonly referred to with terms such as ‘coherence’ [14,26], ‘synergy’ [26,36], ‘integration’ [2,26,31], ‘collaboration’ [32,47], and ‘coordination’ [47]. The term linking is used in order to:

- Avoid challenges and contentions in the differing opinions on how these links take shape, such as those surrounding the suggested absorption of one strategy by the other through integration [20,22,33,36,38,48,49,50];
- Recognize that the links between DRR and CCA may occur differently between, and within, specific contexts such as partial integration on an international level [26], or mainstreaming on a national level [51];
- Acknowledge that not all areas of work in DRR and CCA overlap, or should be linked;
- Provide an opportunity to explore more than the integration of policy or institutions, such as the exploration of technical, operational and conceptual links in implementation.

To provide a background to why linking is important, and the state of the knowledge in this area, we first discuss how these two fields have evolved to address climate change and climate-sensitive disaster risks in health. Next, the state of the art of current literature in linking DRR and CCA in health is presented under the four themes that have emerged from the review. Finally, the article discusses gaps in the literature and considerations for future research.

## 2. Disaster Risk Reduction and Health

Within the context of this review, Disaster Risk Reduction (DRR) is defined as policies and actions developed and implemented with the aim of reducing and preventing disaster risk [52]. Health practitioners have played a pivotal role in responding to health needs in emergencies and disasters, such as disease outbreaks and epidemics including smallpox or HIV/AIDs, natural disasters and human conflict, long before the establishment of health preparedness, response, risk reduction or risk management approaches [53]. Originating from disaster epidemiology, disaster medicine, and emergency preparedness and response in health [54], DRR in health has undergone considerable transformation in the last two decades. This has been spurred on by several events of global concern that have posed significant threats to health, including the September 11 terrorist attacks, and the Ebola outbreak of 2015 [55,56,57,58,59]. One key development of this field in the last two decades is the ongoing shift from a response and preparedness focus, such as through Public Health Emergency Preparedness and Response approaches, to an upstream approach of prevention and recovery [14,15,60,61,62,63]. A second key development is the increasing recognition of the role and importance of health in international DRR policy in the last decade [13,18].

The first international policy framework in DRR, the *Hyogo Framework for Action 2005–2015* (HFA), made little reference to health [16,19,40]. However, the *Sendai Framework for Disaster Risk Reduction 2015–2030*, which superseded the HFA, recognised health as a key contributor to, and beneficiary of, DRR [13,18]. It did so through numerous direct references to health, the expansion of the definition of disasters to include those caused by health-specific hazards (e.g., biological hazards and epidemics), direct reference to the International Health Regulations, and the recognition of health as a cross-cutting issue captured within five of the seven global DRR targets [13,14,16,18,27,40,64,65].

Amidst the increased role and recognition of health in DRR there is a growing body of literature calling for DRR in health to work more closely with actors across other public health and global health initiatives [64,66,67,68]. For example, decreasing the visible separation of actors advocating for the International Health Regulations and Sustainable Development Goals, and the separation of DRR and DRM in health actors from the broader humanitarian system [64]. Public health professionals have a stake in addressing both the health outcomes of a disaster and broader health determinants that may also impact health (e.g., loss of livelihood, environmental damage, and disruption to socio economic services) [16]. Thus, they have a role in broader sustainable development processes. Increasing coherence between these processes is essential to enhance the role of health and collaboration across global health initiatives to address health risks in an all-hazards context, including climate-sensitive disasters and climate change.

## 3. Climate Change Adaptation and Health

Climate Change Adaptation (CCA) refers to “the process of adjustment to actual or expected climate and its effects” and includes moderating or avoiding harm, as well as making the best use of beneficial opportunities [3] (p. 5). Health and other important health-affecting sectors were not featured in the objectives of the Intergovernmental Panel on Climate Change (IPCC) and United Nations Framework Convention on Climate Change (UNFCCC) when they were first established [69]. However, CCA and health has gained momentum since the mid-2000s [1,69,70]. The growth of research and action in climate change and health has been illustrated and spurred on by the IPPC Assessment Reports, which have featured a chapter on health since the Second Assessment Report in 1995 [69]. International policy processes have also been crucial in promoting the importance of both health and adaptation in climate change action, such as National Adaptation Plans (NAPs) and the Lancet Countdown on Climate Change and Health [1,71,72].

The health chapters of the IPCC Assessment Reports Two through Five have shown an increase in understanding in the direct and indirect impacts of climate change on human health [69]. This is particularly illustrated in the case of climate-sensitive diseases and climate-sensitive hazards and extremes. It is noted that there have been challenges with confidently projecting the health impacts of climate-sensitive hazards and extreme events due to the complexity of the pathways leading to health impacts [69]. However, the IPCC 5th Assessment Report, as well as the *Special Report Managing the Risks of Extreme Events and Disasters to Advance Climate Change Adaptation* (SREX) have illustrated that climate-sensitive hazards are likely to increase with the potential for greater health impacts [2,3]. The IPCC SREX was a key publication building clear relationship between disasters and climate change, and calling for linkages between DRR and CCA.

Implementation of CCA in health has largely been driven by the development of NAPs in developing countries. NAPs assist countries to build a greater understanding of climate change risks, vulnerability and adaptation options [71,72]. They also help countries to prioritise and implement adaptation strategies [71,72]. The health component of the NAP (H-NAP) aims to address determinants of health and upstream risk drivers through a health system strengthening approach [71,72]. The H-NAP includes an adaptation plan for health based on a vulnerability and adaptation assessment and outlines specific adaptation goals and mechanisms to achieve these [73]. The NAP process has been, and will continue to be, critical in the effort to increase concrete implementation of CCA in health [73,74]. The development of adaptation plans for health (including H-NAPs), and the integration of these plans into the broader NAP processes is an important indicator used by the Lancet Countdown for Climate Change and Health to track the progress of adapting to indirect impacts of climate change [1].

Tracking the progress made towards CCA for health began with clear and public advocacy by the Lancet Commission on Climate Change and Health in 2015 on the need for health to play a bigger role in climate action [9]. The Commission recognized climate change as an opportunity for furthering health action [9]. However, more engagement from the health sector is needed [9]. To support the increasing role of health, the Commission committed to establishing the Lancet Countdown on Climate Change and Health, an annual report tracking implementation of the health aspects of the Paris Agreement [9]. The first report proposes indicators for monitoring adaptation progress in health [1]. Among these are health impacts and exposure to climate-sensitive hazards including heatwaves, flood and drought [1]. Recently there has been significant progress towards health action for climate change and in the development of understanding and evidence of climate change impacts on health [1]. However, there is significantly less climate change literature relating to health compared to other sectors such as agriculture or tourism [70,75].

Research in CCA in health is still dominated largely by the quantification of risks, identifying vulnerabilities within populations, and projecting changes to health risks. However, there remains a need for this data and information to be accessible, understood and used by policy and decision-makers [74]. There is little research measuring the effectiveness, appropriateness, and comprehensiveness of adaptation action for health [69,76,77], particularly with regards to implementation and capacity development [69,75,77]. Furthermore, there is a need for research on the interaction between CCA in health and other global processes, such as DRR [69]. The following sections discuss why linking DRR and CCA is particularly important in health, and how these links can be made.

## 4. Key Themes from the Literature on Linking DRR and CCA in Health

This review takes a narrative approach in the presentation of the findings. Literature was primarily sourced from Scopus, Web of Science, PubMed, and Google Scholar using ‘disaster risk reduction’, ‘climate change adaptation’ and ‘health’ as search terms. Literature considered relevant to this review needed to explicitly refer to building integration, links, synergies or coherence between DRR, CCA and health in the body of the text (Table 1). The review also draws on integration literature not specific to health where relevant.

The knowledge around why and how to build links between DRR and CCA is growing conceptually, and at international and national policy levels. However, the development of links between DRR and CCA is still in its infancy [26]. Further exploration into how these links are developed in different contexts at various scales is needed, particularly in health. While there has been some discussion on theoretical ways in which DRR and CCA can be linked in health, this knowledge base is scattered and thin. To build a coherent foundation for driving links in health this review summarises the current state of knowledge.

Of the identified literature, five articles refer directly to how DRR and CCA could possibly link in health [15,45,61,80,82]. Three of four articles present possible ideas for building linkages, including: public health preparedness and response as adaptation to climate change; examination of health impact pathways; the concept of vulnerability reduction; and strengthening health systems as no-regrets adaptation [15,61,80]. The other advocates for greater collaboration around data collection to attribute climate change to disaster-related health impacts [82]. None of these draw on primary empirical data to examine these suggestions in practice. Nor is there any subsequent work building on these suggestions, thus the ideas presented in these articles remain theoretical.

Since 2015, there have been an increase in explicit calls for building the coherence between DRR and CCA in the context of health [17,18,40,45,46]. However, the majority of articles are discussion pieces. Only two articles and the book chapter draw on case studies from secondary literature as examples to illustrate points for linkage [17,45,81]. This review revealed no articles which draw on empirical methods to explore why linkage is important in health, or how the links could occur. The key messages drawn from the articles above are discussed in the following sections.

### 4.1. Need for Linking DRR and CCA in Health Will Continue to Grow

The push for greater health involvement in DRR and CCA is highlighted in both areas of global policy and research literature [10,14,16,40,41,44]. The need for linking DRR and CCA in health will increase as the role of health in DRR and CCA continues to grow. As the Sendai Framework has broadened the definition of hazards to include epidemics and pandemics as part of an all-hazard approach to DRR [6,13,14,16,40,44], climate-sensitive diseases emerge as a potential area for overlap between DRR and CCA in health. Furthermore, some categories of climate change impacts, such as heatwaves, are not often classified as hazards associated with disasters within climate change discourse [83,84,85,86,87]. As DRR has expanded the definition of disasters to now incorporate slow-onset stressors such as drought there will be increasing overlap in the implementation of DRR and CCA [88]. These potential areas for additional overlap between DRR and CCA in health have not previously been considered in the literature [88]. These overlaps have the potential to increase as DRR continues to move toward an approach which aims to address underlying risks, just as CCA seeks to address underlying vulnerabilities.

### 4.2. Maximising Conceptual Synergies

Broader DRR and CCA literature indicates that there are overlaps in DRR and CCA strategies [26,29,32,34,47,89,90]. Overlaps also exist in DRR and CCA approaches in health, particularly due to the similar health impact pathways of climate change and climate-sensitive disasters [4]. Anchoring DRR and CCA on conceptual synergies will be important in reaching shared goals of reducing long-term risks [24,26]. Furthermore, maximising conceptual synergies will significantly reduce duplication, and improve the effectiveness and efficiency of implemented strategies [2,8,21,23,24,25,26]. Conceptual synergies are the concepts prevalent in both DRR and CCA in health which can provide conceptual foundations for joint action. Potential conceptual synergies in health include resilience, vulnerability and risk [2,15,17,26,61,80].

Resilience inevitably forms part of DRR and CCA discourse in the context of development and humanitarian aid [37,91,92]. Both approaches work towards resilience as a common aim, therefore meaningful and systematic engagement with resilience is necessary [21,24,25,26,29,35,37,93]. This has also been recognized in the literature on linking DRR and CCA in health [61]. However, resilience is known to have many definitions [94,95,96,97,98] and the utility of resilience as a bridging concept has been contested [99].

Similarly, meaningful engagement with the concepts of risk and vulnerability occur in both DRR and CCA, and these conceptual overlaps offer more motivation to bridge DRR and CCA to address these common aims [38,47,100,101]. Reducing vulnerability is important in addressing the health impacts of both disasters and climate change [15]. Risk and vulnerability reduction are cross-cutting issues in both DRR and CCA in health [15,17,61,80]. The concepts of vulnerability and risk link DRR, CCA and public health practice in multiple ways. First, health emergency preparedness and response has been discussed as reducing climate change vulnerability and disaster risk [15,61,80]. Second, health status has been highlighted as a contributor to disaster and climate change vulnerability and risk [5,9,100,102]. Finally, the determinants of vulnerability to disasters and climate change are similar to the determinants of the health approach commonly used in public health [103]. Incorporation of the social determinants of health has been suggested as a potential opportunity to strengthen both DRR [103] and CCA, particularly if used in conjunction with an iterative approach to decision-making [104,105]. Therefore, conceptual synergies in addressing underlying risks and vulnerabilities are motivators for linkage. Further to this, anchoring DRR and CCA in health on the social determinants of ill-health and vulnerability has been suggested as a potential strategy for building synergy in addressing climate change risk over the long-term [5,6,7]. This is particularly important for addressing indirect health impacts, such as those resulting from food and water insecurity [106,107].

As DRR and CCA continue to become more concerned with building resilience, and reducing underlying risks and vulnerabilities, DRR and CCA will have greater conceptual overlaps [2,35,88]. Resilience, risk and vulnerability concepts present potential opportunities for building conceptual synergies between DRR and CCA in health. However, the utility of these concepts to link DRR and CCA in health is still unknown. Further exploration is needed to understand how these concepts serve as potential joint pathways for linking DRR and CCA and thus reducing health impacts.

### 4.3. Maximizing Technical and Operational Synergies

Linking DRR and CCA is important to build on existing methods and resources in DRR and CCA in order to maximize technical and operational synergies. Technical and operational synergies are those areas of work which offer potential for joint strategies to reduce duplication and strengthen tools, expertise and implementation of DRR and CCA. This shared learning will strengthen the rigor, robustness, effectiveness and efficiency in addressing climate change and disaster risks long-term [16,60,63,80,108,109]. For example, informing disaster risk with the latest climate knowledge and adaptation expertise has been suggested in broader DRR and CCA strategies and is also applicable in health [5,86,110,111]. This is particularly pertinent as DRR needs to shift to a long-term risk prevention approach to address the short- and long-term risks associated with climate change [5,6,16,60,63,80,108]. Similarly, linking DRR and CCA within the health sector can maximize operational synergies by reducing the burden of implementing DRR and CCA separately, including duplicated human and financial resources, particularly in resource constrained contexts [9,14,25,26,36,40,82,110]. Technical and operational synergies are presented together here as often the implementation of DRR and CCA (the operational aspect) cannot be separated from the tools and expertise behind DRR and CCA action (the technical aspect). Some opportunities identified in the literature for technical and operational synergies include: synergies in policy; joint risk and vulnerability assessments; health system strengthening and resilience of health systems; Early Warning Systems (EWS) and risk communication; as well as disaster preparedness and response.

Coherence between national policies are essential for working towards shared goals limiting conflicting or maladaptive strategies. National policy processes, such as the Joint NAPs of several countries in the Pacific, are important for identifying disaster and climate change vulnerabilities, and DRR and CCA options and priorities [26,90]. Numerous examples for successful integration have been identified, including in the South-West Pacific, particularly the Solomon Islands and Vanuatu [51]. These links between DRR and CCA have since been reflected at the regional level through the replacement of the Pacific DRR and Disaster Management Framework for Action 2005–2015 and Pacific Islands Framework for Action on Climate Change 2006–2015 [51] with the Framework for Resilient Development in the Pacific An Integrated Approach to Address Climate Change and Disaster Risk Management 2017–2030, and the development of the Pacific Platform for Disaster Risk Management and Climate Change [112]. These policies themselves represent technical synergies. They also have the potential to facilitate further technical and operational synergies in the implementation of DRR and CCA. National DRR and CCA in health policies are an important foundation for building technical and operational links. The H-NAP process or similar disaster policy and planning processes in health may also offer opportunities for building technical and operational synergies. It is important for climate policies in health to pay adequate attention to the health risks of extreme weather [81]. However, there is no research on links between DRR and CCA through similar policy processes in health. An important step in the H-NAP process is the Vulnerability and Adaptation Assessment.

Risk and vulnerability assessments are important in assessing disaster and climate change risks to the health system and health of populations [16,45,80]. Failing to consider increasing intensity and frequency of disasters as a result of climate change has the potential to lead to maladaptation [36,113,114,115]. The consideration of long-term climate change risk and vulnerability is essential in understanding long-term risks to health. However, joint risk assessments are noted as being rare [45]. Uncertainty and a lack of comprehensive climate change data, as well as little research of climate change on health systems, present significant challenges to joint assessments [45]. As such, a greater understanding is needed on how to conduct joint risk assessments in the context of uncertainty of climate change data, and health and health system impacts. The consideration of health impact pathways is a potential starting point for building technical and operational synergy to address commonalities in health risks of climate change and climate-sensitive disasters [80].

The consideration of health impact pathways has been highlighted as an important and useful way to examine underlying vulnerabilities to climate-sensitive disasters and climate change [80]. Focusing on the pathway between a climate-related event or hazard, and the health outcome, and performing integrated risk assessments, enables health professionals to identify key causes of climate change and disaster risks in health simultaneously and identify opportunities for intervention, including necessary broader approaches at systems level [45]. This presents a potential method for identifying common disaster and climate change vulnerabilities related to health, as well as technical and operation synergies in risk reduction and adaptation strategies. Few [80] states that interventions to reduce health risks of climatic hazards occur at multiple points along the health impact pathway, with the understanding that a holistic and long-term approach is needed in reducing risks. For example, Few [80] suggests that climate change mitigation action even be incorporated in the reconstruction of health facilities post-disaster, as well as the consideration in long-term changes in hazard characteristics resulting from climate change. Therefore, when considering an impacts pathways approach, there is an opportunity for health professionals to consider the full spectrum of upstream to downstream risk reduction and adaptation actions. Thus, this potentially creates greater opportunities for identifying strategies for long-term proactive risk reduction and adaptation [29,37,95,116,117] and maximizing the technical and operational synergies between them. However, this sort of process remains theoretical and its utility has not yet been examined through primary research.

Health systems strengthening has been suggested as an important no-regrets strategy for linking DRR and CCA in health [45]. Health systems strengthening has become an important approach in DRR, particularly since the Ebola outbreak, which forced health practitioners and researchers to examine how resilient health systems are in the face of growing global challenges [64,111,118,119,120,121]. Similarly, the health systems approach has been picked up in CCA and health literature and technical guidance, such as the aforementioned H-NAPs [71], as well as the World Health Organization’s *Operational framework for building climate resilient health systems* [6]. If health systems strengthening is going to be developed as a sustainable long-term solution to disaster and climate change risks in health, joint action will be needed in identifying risks and priorities for the health system. This may include the use of key climate change and health data in health planning to prevent slow-onset health disasters from emerging due to changing pressures on health systems. For example, having an understanding of climate change-related migration can improve the ability of health facilities to accommodate population surges in areas as a result of climate related-disasters and thus improve the resilience of the health system [110]. However, there is little health systems strengthening research that maximizes technical and operational synergies by drawing on both DRR and CCA literature and approaches outside of strengthening health infrastructure. 

Building the resilience of health infrastructure to disasters is relatively well established within DRR. There is technical guidance on the prevention and preparedness of health facilities dating back to the 2000s [122,123,124], and a baseline of technical knowledge and experience has been developed through an iterative process to increase the comprehensiveness of strategies [125,126,127]. Some approaches to building resilience of health facilities also incorporate CCA and mitigation [128]. For example, the consideration of current and future risks exacerbated by climate change in the reconstruction of New York city hospitals and health care system following hurricane Sandy [129,130,131,132]. However, the extent of use of these approaches is currently unknown. Commonly the climate change literature refers to ‘climate-proofing’ health infrastructure [69,74] with little mention of how this fits in with existing DRR efforts. However, there are examples of where this is becoming less prevalent, such as the SMART hospital initiative which offers guidance on how to combine infrastructure resilience with reducing carbon emissions in hospitals [128]. Recent technical guidance for DRR in Hospital facilities, known as the Safe Hospitals Initiative, also includes resilience to future risks and specifically names climate change as key for consideration [125]. It is important to build on the technical expertise developed in both of these approaches to maximize synergies in implementation. However, the application of this in middle and low-income countries is underexplored.

EWS are an important aspect of the resilience of health systems and populations. Both DRR and CCA in health promote EWS as essential tools for preparing for disasters and preventing poor health outcomes from hazards and climate-sensitive diseases [3,23,133]. DRR systems in and outside health have been developing these systems for four decades [23,133]. These efforts have been strengthened by the objectives of the HFA [134], and the Sendai Framework [10]. Recently, a UNFCCC report on adaptation in health highlighted the need to increase risk communication to the public, and early response for climate change, particularly the “need for more guidance to be provided to the public on how to act in the event of climate change impacts such as heatwaves and storms” [74] (p. 18). EWS, public communication strategies, and preparedness for heatwaves are areas that DRR actors in health have existing experience, tools and technical knowledge [83,87]. It is important to draw together DRR and CCA approaches in these areas to ensure they are informed by the most recent climate change science, and early warning and risk communication expertise. Doing so will also be essential to avoid creating unnecessary technical and operational duplication. However, there is no literature relating to how the seemingly separate initiatives do, or will, link together to draw on potential synergies.

Disaster preparedness and response activities in health, including Health Emergency Management, Public Health Emergency Preparedness, among others, have been recognized as adaptation action for climate change [15,61]. By preparing for, and responding to, extreme events and climate-sensitive hazards, countries and populations reduce the potential immediate health risks posed by these events. However, the utility and use of climate change data in preparedness and response is under explored.

The operational synergies of the above suggested synergies exist in the commitment and maintenance of human and financial resources dedicated to the implementation of joint DRR and CCA action in health. There has been wide recognition of the operational needs to link DRR and CCA in the context of the health sector [9,26,40,82,110]. Increased synergies across DRR and CCA in health are suggested to improve funding opportunities [40], as well as the potential for joint policy and capacity development with cross-sectoral engagement [13]. The seven areas of potential technical synergies discussed above—joint policy processes; health systems strengthening; consideration of health impact pathways for identifying opportunities for joint intervention; risk and vulnerability assessment; resilience of health infrastructure; EWS and risk communication; and disaster preparedness and response—are also opportunities for potential operational synergies.

It is essential to build on existing knowledge and expertise in each field in order to strengthen the approaches in both fields. However, as outlined here, there are several areas where the synergies in complementary knowledge, expertise and tools are not being maximized. Currently there is a paucity of research surrounding technical and operational synergies that are or could be made between DRR and CCA in health. Moreover, a significant knowledge gap exists on the nature of these technical synergies and how to build them.

### 4.4. Public Health Actors: Critical in Bridging DRR and CCA

Practitioners and administrators play an important role in bridging DRR and CCA [33]. Practitioners and administrators working towards improving and securing population health will be vital contributors to effective implementation of DRR and CCA strategies [61]. These public health professionals are experienced in dealing with risk. It has been suggested that public health professionals could thus be readily familiar with concepts and approaches that are commonly used in DRR and CCA [61,67,103,135,136]. Additionally, the public health sector is experienced in addressing complex problems through multi-sectoral action [137]. As a result, public health professionals are in a strong position to establish and sustain links between and bridging DRR and CCA in health.

#### 4.4.1. Understanding of Central Concepts and Approaches

Public health professionals are experienced in dealing with risk, as well as vulnerability (created by the social determinants of health) in their day to day work functions. Thus, there is the potential for public health professionals to have a strong understanding of resilience, vulnerability and risk [67,103,135,136]. Resilience has been identified as a useful and relevant conceptual framework in DRR approaches in health to align day to day public health activities with DRR and DRM activities in health, allowing public health professionals to begin to see themselves as both public health and DRR practitioners [67]. As such, the familiarity with the concept of resilience may also aid public health professionals with identifying tangible links between DRR and CCA strategies in health.

Commonalities have also been drawn between the concept of vulnerability and the determinants of health in disaster and climate change literature [67,103,135,136]. Public health professionals are inherently familiar with, and have an understanding of, the various causes of underlying risks, including vulnerabilities and complex interactions of determinants of health, including in the context of climate change and disasters [17,67,74,103,104]. This familiarity and experience are useful for interpreting and addressing the direct and indirect health impact pathways, and simultaneous underlying disaster [67] and climate change risks [104]. Furthermore, public health professionals are accustomed to addressing similar types of risk through preventative strategies [63]. Population/public and primary health services work across scales, with a significant focus on communities and vulnerable groups [103]. As such, public health professionals are accustomed to varying risk profiles within populations, as well as addressing health vulnerabilities and needs in complex socio-ecological systems [15]. The unique positioning of the health sector within the community has the potential to improve implementation of CCA and DRR strategies in health as local-level action is important within complex multi-level adaptation and risk reduction [15,61]. Public health professionals are also experienced in engaging and empowering communities to appropriately address their needs [138]. Thus, public health professionals are well-positioned to understand and address the simultaneous climate change and disaster health risks that communities and vulnerable populations face.

#### 4.4.2. Experience in Addressing Complex Problems through Multi-Sectoral Action

Climate change and disasters present significant health risks which occur through complex social and environmental pathways, affecting multiple systems simultaneously [1,2,45]. Addressing these risks will require public health professionals and administrators to draw on previous experience addressing complex public health challenges. Public health challenges often stretch across multiple sectors outside of the health sector alone, and require systemic, long-term solutions, such as when addressing obesity or tobacco control [139,140]. Therefore, public health professionals are experienced in dealing with long-term risks [71] fraught with uncertainty such as those that exist in models of climate change impacts on health [71,104,105], as well as health issues which require multi-sectoral action [103,137,140]. Public health has strengths in adopting socioecological system-wide perspectives, understanding complex interactions in health impact pathways, and developing multi-dimensional strategies for intervention that incorporate the needs and views of key stakeholders including communities and policy makers [103,140]. The strength of health in multi-sectoral action is essential for developing solutions to the complex problems presented by the health risks of disasters and climate change [40,141]. Within this context, public health professionals and administrators working in DRR and CCA should be well equipped to strengthen coordination and develop cross-cutting solutions to address these complex challenges [64,141].

#### 4.4.3. Strengthening Collaboration

While recognizing the strengths of health professionals to potentially build these links, it is also important to acknowledge the silos that exist between groups working on DRR and CCA in health [49,57,67,142]. Therefore, greater collaboration and coordination will be necessary between pertinent groups working on DRR and CCA in health [26,40]. This has been called for in surveillance of post-disaster health impacts and evaluation of the effect of climate change on disaster-related health impacts [82]. However, collaboration needs to extend beyond attributing the climate change component of disaster-related health impacts to enable DRR and CCA to be linked. Collaboration on DRR and CCA to address social determinants through inter-sectoral coordination with health-affecting sectors has been suggested as important [14,17,78,80].

## 5. Conclusions

Linking DRR and CCA in health is critical for effectively addressing the numerous health impacts of disasters and climate change. As the role of health grows in both DRR and CCA, the need for linking these two approaches will increase. Linking DRR and CCA in health is fundamental to maximise conceptual linkages in order to reach common goals including building resilience, and reducing risk and vulnerability. Therefore, there is a need for empirical research to investigate if and how these common concepts can facilitate linkages between DRR and CCA in health. Similarly, linking these two fields can build technical and operational synergies. So doing builds on: existing expertise and resources available in each field, including in policy; health systems strengthening; risk and vulnerability assessment; resilience of health infrastructure; EWS and risk communication; disaster preparedness and response; and the examination of health impact pathways to identify points for joint intervention. Health professionals and administrators play a vital role in building these links due to their experience in dealing with risks and complex health problems, and potential familiarity with concepts and approaches in DRR and CCA. However, there is currently a lack of in-depth knowledge on how these links can be advanced and the forms they take, as well as the opportunities, challenges and enablers for building and sustaining these links. Therefore, empirical examination of the development of links between DRR and CCA in health is necessary in order to understand how best to address the growing health risks from climate change and climate-sensitive disasters.

## Figures and Tables

**Table 1:** Key messages of articles on linking DRR and CCA in health.

Article	Type	Aim	Key Messages on Linking DRR and CCA in Health
Aitsi-Selmi et al. (2015) [40]	Review	Review of health in Sendai Framework process.	Increasing coherence in global policy on DRR, CCA and sustainable development. Sendai Framework highlights that health is key in DRR, and synergy is important.
Aitsi-Selmi and Murray (2015) [13]	Editorial	Narrative review of health in Sendai Framework process.	Calls for better coherence and recognition of health across DRR, Sustainable Development Goals and climate change policy.
Aitsi-Selmi and Murray (2015) [14]	Special Report	Review of health in Sendai Framework process.	Post-2015 policies are an opportunity for global policy coherence. Health is of growing importance in DRR on a global level.
Aitsi-Selmi et al. (2016) [78]	Review	Review (not health focused) of science and technology in DRR in relation to the Sendai Framework.	Increasing linkage in policy relating to DRR, development, health and climate change occurred through the 2011 and 2013 Global Platforms for DRR. Recognises an opportunity for collaboration on science and technology in risk assessment and early warning which will influence DRR, climate change, agriculture, healthcare, etc. Acknowledges the need to recognise different agendas of each group and negotiate mutual ways forward.
Aitsi-Selmi, Murray and Wannous (2017) [17]	Book chapter	Review of health impacts; and synergy of both health and climate change, and health and disasters.	Post-2015 policies are an opportunity for building policy coherence. Suggests that health is a point of convergence for DRR, CCA and sustainable development. Social determinants of health important in acting on synergies (e.g., strengthening nutrition services; addressing obesity through women’s education; and place-based initiatives such as Healthy Cities).
Banwell et al. (2018) [4]	Commentary	Reviews common pathways of health impacts.	Commonalities in health impacts of climate change and climate-sensitive disasters as a first step for developing a common language for joint action.
Bowen and Friel (2012) [79]	Review	Overview of adaptation and how it relates to improving health and reducing health inequities.	Discusses the need for synergy between CCA and sustainable development in addressing social determinants of health and then goes on to mention disaster management as an important partner. CCA is an opportunity to improve synergies across disciplines that contribute to health. Strategies that address the social determinants of health contribute to sustainable development.
Few (2007) [80]	Theoretical framework	Theoretical analysis of social science concepts and approaches relating to health impacts of climate hazards.	Health impacts pathways suggested as a method for identifying points of intervention. Reducing risks should be focused on breaking cycles of vulnerability and adapting to changes in hazards and the population. Example strategies include supply of vessels for water storage and treatment, sanitation facilities, community-based health volunteers, stock piling emergency medicines, hospital disaster plans, location of health facilities.
Filho et al. (2018) [81]	Review	Provides examples from eight countries to examine the health impacts of climate risks and extreme events.	Climate policies that adequately address extremes. Suggests joint resource mobilisation for addressing climate risks and extreme events
Hashim, J.H. and Hashim, Z. [46]	Review	Review seeking to associate climate change with extreme weather impacts on health in Asia.	Calls for further assessment of health impacts of climate change and extreme weather events in Asia.
Keim (2008) [61]	Discussion	Theoretical discussion of the role of preparedness and response in reducing vulnerability to climate change.	Suggests public health preparedness and response as an adaptation to climate change. Vulnerability reduction reduces susceptibility and increases resilience to disasters and climate change.
Keim (2011) [15]	Discussion	Theoretical discussion of DRR in health as sustainable CCA.	Proposes a comprehensive approach to reducing vulnerability to disasters as adaptation to climate change. Calls for the mainstreaming of CCA, DRR and public health in activities of health affecting sectors and sustainable development.
Phalkey and Louis (2016) [45]	Review	Reviews disaster and climate change impacts on health and calls for simultaneous action	Calls for systematic preparedness and adaptation strategies that address both disaster and climate change risks simultaneously. Joint impact assessment rarely occurs. Health systems strengthening as a ‘no-regrets’ strategy.
Sauerborn and Ebi (2012) [82]	Review	Reviews findings of the SREX in an attempt to attribute disaster-related health impacts to climate change.	Links climate change and disaster-related health impacts. Calls for greater collaboration between climate scientists, health researchers, policy makers and disaster community, particularly on post-disaster surveillance and data collection for attributing disaster-related health impacts to climate change. Provides a conceptual framework on the link between climate and health-related disasters based on an expanded version of the SREX.

DRR: policies and actions developed and implemented with the aim of reducing and preventing disaster risk [52]; CCA: “the process of adjustment to actual or expected climate and its effects” [3] (p. 5).; SREX: Special Report Managing the Risks of Extreme Events and Disasters to Advance Climate Change Adaptation [2].
